# Targeting the JAK/STAT pathway with isoliquiritigenin in ovarian cancer: molecular mechanisms and therapeutic implications

**DOI:** 10.3389/fphar.2026.1828829

**Published:** 2026-06-04

**Authors:** Meidan Ding, Fangfang Guo, Ping Lin

**Affiliations:** Affiliated Xinhua Hospital of Dalian University, Dalian, China

**Keywords:** isoliquiritigenin, JAK/STAT signaling pathway, molecular mechanisms, ovarian cancer, therapeutic potential, tumor immune microenvironment

## Abstract

Ovarian cancer, one of the most common gynecological malignancies, ranks as the fifth leading cause of cancer death in women. To investigate the mechanism by which isoliquiritigenin (ISL) regulates the JAK/STAT signaling pathway in ovarian cancer intervention, this review integrates cellular, animal, and preclinical studies on ISL treatment for ovarian cancer, explores how ISL modulates the JAK/STAT pathway and affects ovarian cancer cell behavior and the tumor immune microenvironment, and summarizes the related mechanisms and research progress. The results show that ISL exerts significant anti-ovarian cancer effects through multitarget regulation of the JAK/STAT pathway. With the advantages of low toxicity and multi-pathway modulation, ISL is a promising natural candidate for targeted therapy, reversal of drug resistance, and combination therapy in ovarian cancer, thus holding great value for basic research and clinical translation.

## 1 Introduction

Ovarian cancer is a heterogeneous malignancy originating from ovarian epithelial-mesenchymal cells or germline cells, with the highly malignant serous subtype being the most lethal (Radu et al., 2021), often accompanied by BRCA mutations and TP53 deletions, and grows insidiously, leading to extensive peritoneal metastasis (Samuel et al., 2022). Standard treatment involves cytoreductive surgery combined with platinum-based chemotherapy, yet recurrence rates remain high with a 5-year survival rate of approximately 50% (Caruso et al., 2025). Silva and Stockman (2024) indicate that the 5-year survival rate for ovarian cancer in the United States increased from 36% in 1975 to 51% in 2019. According to GBD 2021 statistics, the age-standardized incidence rate of ovarian cancer among women aged 15–24 worldwide was 1.85 per 100,000 in 2021, with an average annual increase of 0.85% from 1990 to 2021. The burden is predominantly concentrated in low- and middle-SDI countries, with 62% of case increases attributable to genuine risk elevation rather than population growth. This underscores that ovarian cancer remains one of the diseases severely impacting human health (Che and Yin, 2025). The JAK/STAT signaling pathway is a central regulatory axis mediating tumor proliferation, invasion, immune evasion, and chemotherapy resistance. It plays a critical role in the initiation and progression of ovarian cancer. Constitutive activation of this pathway promotes unlimited proliferation of ovarian cancer cells, suppresses apoptosis, accelerates tumor microenvironment remodeling, and exacerbates peritoneal metastasis and chemotherapy resistance. These effects contribute to treatment bottlenecks, high recurrence rates, and poor prognosis in ovarian cancer (Wu et al., 2019). Therefore, targeting aberrant JAK/STAT activation has become a promising research direction for targeted intervention and adjuvant therapy of ovarian cancer. Isoliquiritigenin (ISL), a natural flavonoid compound extracted from licorice, has shown significant research progress in ovarian cancer treatment in recent years. ISL inhibits tumor growth by downregulating the Akt-Wee1-CDK1 signaling pathway (Zhou et al., 2023; Li et al., 2025) and by modulating the PI3K/AKT/mTOR pathway to influence the interplay between autophagy and apoptosis (Zhang X. R. et al., 2018). These combined mechanisms impair proliferation and increase apoptosis in ovarian cancer cells, resulting in significant antitumor effects. Stanilov et al. (2024) demonstrated that persistent abnormal activation of the IL-6/JAK2/STAT3 pathway significantly drives ovarian cancer cell proliferation, anti-apoptosis, and epithelial-mesenchymal transition (EMT). Overactivation of this pathway accelerates the G1/S phase transition of ovarian cancer cells and reduces the level of apoptosis. In targeted intervention studies of this pathway, ruxolitinib, a selective JAK1/2 inhibitor, competitively binds to the ATP-binding site of JAK kinases, thereby efficiently blocking STAT3 phosphorylation and nuclear translocation, and subsequently inhibiting STAT3 pathway activation. In contrast, ISL suppresses STAT3 tyrosine phosphorylation by interfering with the crosstalk between the TGF-*β*/Smad and JAK/STAT pathways, while also downregulating the anti-apoptotic protein Bcl-xL and matrix metalloproteinase MMP-9. Although ISL is less effective than ruxolitinib in inhibiting ovarian cancer cell proliferation, it offers the advantages of natural origin, low toxicity, and broad-spectrum activity. By exerting anti-tumor effects through synergistic multi-pathway regulation, ISL holds significant potential for reversing the malignant biological phenotype of ovarian cancer.

## 2 Chemical structure and biological activity of ISL

### 2.1 Chemical structural characteristics of ISL

ISL is a chalcone compound with the chemical name 1-(2,4-dihydroxyphenyl)-3-(4-hydroxyphenyl)-prop-2-en-1-one, with the molecular formula C15H12O4 and molecular weight 256.25 (Mustafa et al., 2025). Liu et al. (2024) characterized the structure via ^1^H NMR, ^13^C NMR, and HRMS, confirming the presence of two aromatic rings (Ring A and Ring B). These rings are cross-conjugated via an acetonone bridge containing an *α,β*-unsaturated ketone structure. Hydroxyl groups are present at the 2′,4′positions of Ring A and the four position of Ring B. The bridging structure contains three atoms with high rotational freedom, enabling the extensive distribution of receptor-binding sites. The chalcone structure of ISL is key to its biological activity. The conjugated double bond system in this structure enhances its antioxidant capacity (Hu et al., 2023) by directly scavenging free radicals, thereby mitigating oxidative stress-induced cellular damage (Egbujor et al., 2021; Mustafa et al., 2025). Regarding antitumor effects, ISL induces tumor cell apoptosis and autophagy via the PI3K/Akt/mTOR signaling pathway (Shen et al., 2024), with the phenolic hydroxyl and ketone groups in its chalcone skeleton serving as crucial structural foundations for this action. ISL exerts biological activity through multiple pathways. Zhang et al. (2020) revealed that ISL acts as an allelochemical in plants, inducing oxidative stress by triggering excessive reactive oxygen species (ROS) production. This leads to membrane lipid peroxidation and cell death, thereby inhibiting plant root tip growth. Regarding neuroprotection, Shi et al. (2020) confirmed that the conjugated double bonds of ISL interact synergistically with 4-hydroxy to scavenge H2O2, upregulate SOD2 transcription, and restore mitochondrial membrane potential. The Bax/Bcl-2 ratio decreased from 10.42 to 0.93, caspase-3 activity was inhibited, and the cell survival rate increased. ISL enhances antioxidant enzyme activity by suppressing ROS production and modulating apoptosis-related gene expression, thereby protecting neurons from oxidative stress damage (Wu et al., 2022; Huang et al., 2024).

### 2.2 Overview of the biological activity of ISL

ISL is mainly derived from the roots and rhizomes of licorice plants of the genus *Glycyrrhiza*, including *Glycyrrhiza uralensis*, *Glycyrrhiza glabra*, and *Glycyrrhiza inflata* (Table 1). It exhibits significant antioxidant capacity, effectively scavenging free radicals and reducing oxidative stress-induced cellular damage. By activating the Nrf2/ARE signaling pathway, ISL induces the expression of antioxidant enzymes such as HO-1 and NQO1, thereby enhancing cellular antioxidant defense systems (Li M. et al., 2022; Qiu et al., 2024). Regarding anti-inflammatory effects, ISL suppresses NF-*κ*B and MAPK signaling pathways, reducing inflammatory factor production and alleviating inflammatory responses (Li M. et al., 2022). Simultaneously, it maintains intracellular antioxidant defense mechanisms, including superoxide dismutase (SOD), catalase (CAT), and glutathione peroxidase (GPx) activity to mitigate oxidative stress-induced cellular damage (Zhang Z. et al., 2022). Its antiviral activity manifests as ISL’s ability to disrupt viral replication cycles, inhibit viral protein synthesis, and suppress multiple viruses. Within the tumor microenvironment, ISL reduces ROS production by activating the Nrf2 pathway or upregulating NADPH oxidase expression and activity (Chen et al., 2012; Alkuwayti, 2025). In SK-MEL-28 melanoma cells, ROS inhibition suppressed p38*α* (Thr180/Tyr182) phosphorylation, blocked the p38-mTOR-STAT3 (Ser727) cascade. This leads to decreased STAT3 transcriptional activity, silencing downstream cyclin D1 and survivin expression, arresting cells at the G2/M boundary, and increasing apoptosis rates (Kwon et al., 2025). Pancreatic ductal adenocarcinoma relies on basal autophagy to maintain oxidative homeostasis. ISL inhibits autophagy flux in pancreatic cancer cells by activating the p38-MAPK signaling pathway, leading to accumulation of autophagosome markers LC3-II and p62 while blocking autophagosome-lysosome fusion. This mechanism disrupts cellular clearance of damaged mitochondria, triggers endoplasmic reticulum stress and oxidative stress, ultimately inducing apoptosis. The chemically synthesized derivative ISL-17 in MKN45 gastric cancer cells interferes with energy metabolism (Warburg effect) by inhibiting GLUT4-mediated glucose uptake, leading to decreased ATP levels and increased ROS accumulation. This process triggers an energy crisis by blocking glycolysis and oxidative phosphorylation, further enhancing the cytotoxic effects of ROS and thereby inhibiting tumor growth (Li et al., 2025). Collectively, these mechanisms confer ISL a triple attack pathway targeting oxidation, metabolism, and immunity, positioning it as a low-toxicity, multi-target candidate molecule for combination therapies against solid tumors.

**TABLE 1:** Natural sources of ISL.

Plant/Herb name	Latin name	Family	Used part(s)	Note
Gancao (licorice)	*Glycyrrhiza uralensis* Fisch.	Fabaceae	Root and rhizome	Main source of isoliquiritigenin; core research object in literature
Guangguogancao	*Glycyrrhiza glabra* L.	Fabaceae	Root and rhizome	Main licorice species in Europe; contains isoliquiritigenin
Zhangguogancao	*Glycyrrhiza inflata* Bat.	Fabaceae	Root and rhizome	One of the licorice origins accepted by the Chinese Pharmacopoeia
Hongqi	*Hedysarum sinense* Oliv.	Fabaceae	Root	Traditional Chinese medicinal herb containing isoliquiritigenin
Huangqi (Astragalus)	*Astragalus membranaceus* (Fisch.) Bunge	Fabaceae	Root	Isoliquiritigenin can be detected in the root
Shizhu (Carnation)	*Dianthus chinensis* L.	Caryophyllaceae	Aerial part	Non-Fabaceae source; contains isoliquiritigenin
Yingzuidou (Chickpea)	*Cicer arietinum* L.	Fabaceae	Seedling, sprout	Edible and medicinal plant source

## 3 Regulatory mechanism of ISL on the JAK/STAT signaling pathway

ISL acts primarily by inhibiting upstream JAK kinase activity and reducing STAT protein phosphorylation, while simultaneously regulating STAT protein nuclear translocation, downstream transcription, and the expression of upstream cytokines and receptors. It also intervenes in pathway activity through multiple interconnected routes. Given that aberrant activation of the JAK/STAT pathway can reshape the tumor microenvironment, ISL helps restore microenvironmental homeostasis by targeting this pathway.

### 3.1 Regulation of JAK kinases by ISL

ISL significantly inhibits the phosphorylation activity of JAK2 by directly acting on members of the JAK kinase family, particularly JAK2 (Zhang et al., 2024). ISL suppresses PGE2 and IL-6 signaling in colitis-associated tumorigenesis by blocking M2 macrophage polarization (Wu et al., 2016). Ganesan et al. (2024b) used a breast cancer bone metastasis model and found that ISL reduces JAK2/STAT5 signaling-mediated tumor cell proliferation and bone resorption. This effect is achieved by downregulating JAK2 expression and decreasing JAK2-STAT5 interaction. In RAW264.7 cells, it reduced JAK2 phosphorylation levels and decreased STAT5 nuclear translocation, thereby inhibiting RANKL-induced osteoclast formation. *In vivo* studies revealed no significant hepatotoxicity or nephrotoxicity in mouse livers and kidneys, indicating good safety at therapeutic doses (Xue and Wang, 2025). Ovarian cancer is prone to peritoneal invasion and distant metastasis, accompanied by inflammation in the tumor microenvironment and abnormal activation of stromal cells. The JAK2/STAT5 pathway is commonly aberrantly activated in ovarian cancer tissues and participates in cancer cell proliferation, microenvironment remodeling, and metastatic regulation. Breast cancer and ovarian cancer are both hormone-related malignancies and share highly similar signaling pathway characteristics. Therefore, the regulatory effect of ISL on JAK2, as confirmed in the breast cancer study, can be directly applied to the molecular intervention needs of ovarian cancer. Additionally, ISL inhibits apoptosis associated with JAK2 and STAT3 in human renal carcinoma (Yin et al., 2024). In the ovarian cancer microenvironment, tumor-associated macrophages (TAMs) and cancer-associated fibroblasts (CAFs) are the main sources of IL-6 secretion, forming an autocrine/paracrine loop that continuously activates the JAK/STAT3 axis. ISL exhibits a particularly strong inhibitory effect on JAK2; this inhibition is not limited to JAK2, as ISL also shows weaker effects on JAK1 and JAK3. However, because JAK2 is the primary target of ISL, changes in its activity have the most significant impact on the overall signaling pathway. Sun et al. (2021) demonstrated significant regulation of JAK kinases in diabetic nephropathy model rats. The study indicated that long-term ISL treatment effectively suppressed activation of the JAK2/STAT3 signaling pathway, specifically by reducing the expression levels of p-JAK2 and p-STAT3 proteins in renal tissue. This regulatory mechanism helps mitigate inflammatory responses in diabetic nephropathy, thereby protecting renal function. In cancer models, ISL reduced the expression of pro-survival genes and induced cancer cell apoptosis by inhibiting the JAK/STAT pathway (Wan et al., 2024); in inflammatory models, ISL alleviated tissue damage and inflammatory responses by downregulating the expression of inflammatory factors (Huang X. et al., 2020).

### 3.2 Regulation of STAT proteins by ISL

ISL precisely regulates STAT protein activity through a synergistic mechanism that inhibits upstream kinase activity, blocks nuclear translocation, and promotes dephosphorylation, thereby intervening in JAK/STAT-mediated pathological processes such as inflammation and proliferation. ISL targets JAK kinases such as JAK1 and JAK2, and by inhibiting their activity, it reduces phosphorylation of STAT proteins such as STAT3 and STAT1 (Prasad et al., 2010). Phosphorylation is a critical step in STAT protein activation, and unphosphorylated STAT proteins struggle to form homodimers or heterodimers (Siering et al., 2022). Second, for phosphorylated STAT proteins, ISL interferes with their nuclear translocation process, preventing activated STAT dimers from entering the nucleus to bind specific DNA response elements, such as STAT3-binding promoter regions (Krajka Kuźniak et al., 2024). This inhibits the transcriptional expression of downstream pro-inflammatory factors, such as IL-6 and TNF-α, and proliferation-related genes, such as Cyclin D1 (Gu et al., 2024). Kim et al. (2017) noted that ISL significantly reduces STAT3 phosphorylation at Y705 and S727, thereby blocking STAT3 activation. Additionally, ISL accelerates the dephosphorylation of phosphorylated STAT proteins by upregulating the activity of protein phosphatases, such as SHP-1 (Bajalia et al., 2022). This facilitates their return to an inactive state and further attenuates the pathway’s abnormal activation effects.

Studies have confirmed that the regulation of ROS by ISL depends on cell type, concentration, time, and pathway, showing bidirectional effects. In normal cells and oxidative injury models, ISL primarily acts as an antioxidant and suppresses ROS accumulation. In contrast, in tumor cells and the antitumor immune microenvironment, ISL promotes ROS burst by interfering with energy metabolism and reshaping immune phenotypes, thereby exerting anticancer effects. Fan et al. (2025), Krajka Kuźniak et al. (2024) reported that ISL significantly suppresses ANXA2 expression, thereby blocking the phosphorylation and activation of STAT3, reducing p-STAT3 levels, and affecting downstream signaling pathways—a key manifestation of its kinase inhibition mechanism. Concurrently, ISL activates the Nrf2 antioxidant pathway, enhancing antioxidant enzyme activity and reducing ROS accumulation. Through a negative feedback mechanism, it further inhibits sustained STAT3 activation. Under normal physiological conditions, redox balance is maintained in cells, and ROS remain at a relatively stable low level. When cells are exposed to external stimuli, ROS levels may rise. Excessive ROS can activate a series of signaling pathways, including those involving STAT3. Nrf2 is an important transcription factor that plays a central role in antioxidant defense. After ISL activates the Nrf2 antioxidant pathway, Nrf2 translocates into the nucleus and binds to the ARE, initiating the transcription and expression of various antioxidant enzyme genes, such as SOD and GPx. These enzymes clear excess ROS and reduce ROS levels. Once ROS levels decline, the STAT3 activation previously driven by ROS elevation is suppressed through negative feedback, thereby preventing STAT3 hyperactivation (Gao et al., 2020). Wang et al. (2019) demonstrated that ISL treatment significantly increased ROS levels in HepG2 cells in a time-dependent manner, while NAC pretreatment reversed this effect, confirming the negative feedback role of ROS in ISL-mediated STAT3 regulation. Furthermore, ISL regulates the methylation and acetylation states of STAT3-related genes through epigenetic reprogramming, thereby suppressing their transcriptional activity (Sun et al., 2021). Cotoraci et al. (2021) reported in multiple myeloma studies that ISL’s regulation of STAT proteins inhibits myeloma cell proliferation. By reducing STAT3 phosphorylation levels, it decreases STAT3 nuclear translocation and DNA binding to proliferation-related genes such as cyclin D1. This causes myeloma cells to arrest in the G0/G1 phase of the cell cycle, inhibiting cell proliferation while simultaneously promoting the expression of apoptosis-related genes and inducing myeloma cell apoptosis.

### 3.3 Upstream receptors and cytokine microenvironment

ISL significantly downregulates the expression of IL-6 receptor (IL-6R), IL-2 receptor *β* subunit (IL-2R*β*), and interferon *α/β* receptor 1 (IFNAR1). This downregulation directly reduces cytokine signaling by blocking cytokine receptor binding, thereby inhibiting downstream inflammatory responses. At the cytokine release level, ISL exhibits dose-dependent and time-dependent inhibition of soluble IL-6, TNF-*α*, and IFN-*β*. Traboulsi et al. (2015) demonstrated in an influenza virus infection model that ISL treatment reduced mRNA levels of TNF-*α*, IL-1*β*, and IFN-*β* by over 60%, an effect partially reversed by the PPAR*γ* antagonist GW9662, suggesting a central role for the PPAR*γ* pathway in ISL-mediated immunosuppression. Further studies have shown that ISL inhibits NF-κB nuclear translocation by activating the PPAR*γ* and Nrf2 signaling axes, thereby attenuating virus-induced inflammatory gene expression.

**FIGURE 1:**
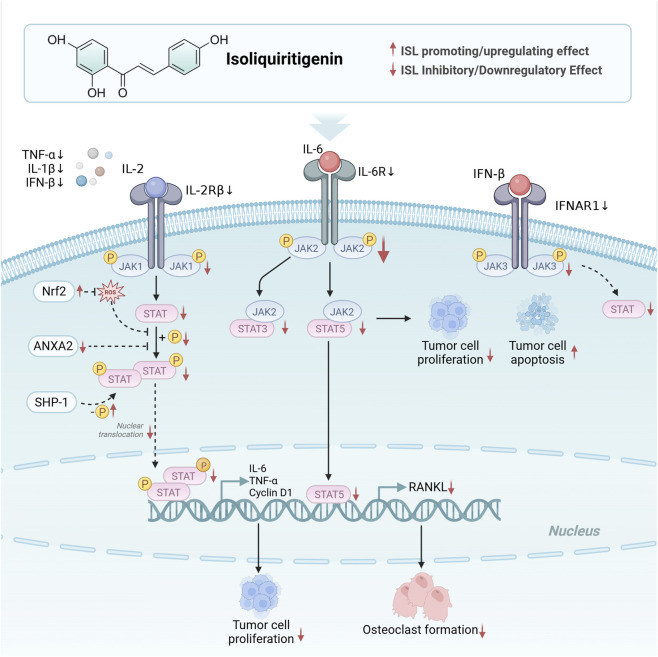
Regulatory mechanisms of ISL on the JAK/STAT signaling pathway. Note: Red upward arrows (↑) indicate ISL-mediated promotion or upregulation; red downward arrows (↓) indicate ISL-mediated inhibition or downregulation. Black arrows indicate signal transduction or regulatory relationships. “P” denotes phosphorylation. ISL directly inhibits JAK1/2/3 phosphorylation, thereby reducing STAT3/5 activation and nuclear translocation. ISL also downregulates cytokine receptors (IL-6R, IL-2Rβ, IFNAR1) and inflammatory cytokines (TNF-α, IL-6, IL-1β, IFN-β), and upregulates Nrf2 and SHP-1 to promote STAT dephosphorylation and antioxidant responses. ANXA2 acts as an upstream facilitator of JAK/STAT signaling. Ultimately, ISL suppresses tumor cell proliferation and osteoclast formation while inducing apoptosis.

In addition, ISL induces HO-1 expression through Nrf2 activation, and the HO-1 metabolites biliverdin and carbon monoxide inhibit TLR7/8-mediated IFN-*β* release pathways (Wang et al., 2024; Yao D. et al., 2024). Baranwal et al. (2021) reported that ISL blocks early IFN-*β* bursts by suppressing the TLR-NF-κB-IRF3 axis in viral infection models, thereby suppressing the secondary amplification of IL-6 and TNF-*α*. ISL downregulation of IL-2R*β* may occur via inhibition of the JAK1/3-STAT5 signaling axis, which weakens Th1 cell differentiation and IFN-*γ* production, thus indirectly reducing IL-6 and TNF-*β* expression levels (Figure 1).

Although the above evidence is mainly derived from viral infection or inflammation models and direct validation in the ovarian cancer microenvironment remains insufficient, given the critical role of the IL-6/JAK/STAT3 axis in ovarian cancer, the modulation of these upstream receptors and cytokines by ISL still holds significant potential. Future studies are needed to further validate these effects in ovarian cancer-specific models.

## 4 Mechanism of action of ISL in ovarian cancer intervention

### 4.1 Inhibition of ovarian cancer cell proliferation and apoptosis induction

In ovarian cancer intervention, ISL effectively inhibits cell cycle progression in ovarian cancer cells by regulating the JAK/STAT signaling pathway (Baranwal et al., 2021). Specifically, ISL reduces JAK kinase activity, thereby decreasing the phosphorylation of STAT proteins and blocking their nuclear translocation (Yin et al., 2023), thereby inhibiting transcription of downstream target genes closely associated with cell cycle regulation. This process downregulates cyclin expression, such as Cyclin D1 and Cyclin E, while upregulating cyclin-dependent kinase inhibitor expression, such as p21 and p27 (Huang Y. et al., 2020). Ultimately, it arrests ovarian cancer cells at the G1/S transition and blocks entry into DNA synthesis, thereby effectively inhibiting cell proliferation. Zhang B. et al. (2018) reported that 25 μM ISL triggered G2/M arrest in SKOV3 and OVCAR5 cells. Flow cytometry analysis revealed a 42% decrease in cyclin B1 protein and a 55% reduction in phosphorylated CDK1 (Thr161), preventing CDC25C from removing the inhibitory phosphorylation of CDK1 and locking cells at the pre-mitotic stage.

At the same concentration, mitochondrial membrane potential decreased by 35% within 8 h, cytoplasmic cytochrome c increased 2.7-fold, Apaf-1 oligomerization enhanced, caspase-9 activity rose 4.1-fold, and executor caspases-3/7 peaked at 24 h, with substrate PARP cleaved by 78%. Rebalancing of the Bcl-2 family was the key initiator: anti-apoptotic Bcl-2 and Mcl-1 were downregulated by 50% and 44%, respectively, while pro-apoptotic Bax translocated to mitochondria and increased 3.2-fold. Bak oligomerization increased 2.9-fold, and mitochondrial outer membrane permeability irreversibly increased. It should be noted that although the above studies provide precise quantitative data, they have certain limitations. The SKOV3 and OVCAR5 cell lines used represent only some subtypes of ovarian cancer cells, making it difficult to generalize the findings to all pathological types. Moreover, differences exist between *in vitro* cell experiments and the *in vivo* microenvironment, and the impact of different experimental conditions on data reproducibility has not been clarified. In addition, the two studies did not perform repeat validation or multi-center experiments, and the sample sizes were limited. Therefore, further high-quality studies are needed to confirm the generalizability of the conclusions. Chen et al. (2019) further revealed that this arrest was accompanied by a 38% decrease in JAK2 phosphorylation levels within 6 h and a 51% reduction in STAT3 (Tyr705) phosphorylation at 12 h; Chromatin immunoprecipitation confirmed that STAT3 could no longer bind the cyclin D1 promoter. Cyclin D1 mRNA decreased by 63% at 24 h, Rb protein remained hypophosphorylated, E2F1 transcriptional activity was suppressed, S-phase entry was blocked, EdU incorporation decreased by 45%, and cell population doubling time extended from 28 h to 52 h. Chen used a constitutively activated STAT3 mutant for transfection, which reversed Bcl-2 downregulation and reduced caspase-3 activity by 60%, confirming the JAK/STAT pathway as the upstream switch for Bax/Bak activation. In the nude mouse peritoneal dissemination model, treatment with ISL at 50 mg kg^-1^·d^-1^ for 21 days reduced tumor nodules from 42 to 11 and decreased ascites volume by 68%. Immunohistochemistry showed a decrease in STAT3 nuclear positivity from 85% to 19%, while TUNEL-positive cells increased 5.7-fold, demonstrating consistent *in vivo* and *in vitro* effects.

### 4.2 Inhibition of ovarian cancer cell invasion and metastasis

ISL achieves precise intervention in the invasion-metastasis cascade of ovarian cancer cells by dual inhibition of core EMT transcription programs and matrix metalloproteinase expression. Ray et al. (2023) systematically reviewed that cytokines such as TGF-*β*, IL-6, and IL-8 sequentially activate the Smad2/3, JAK2/STAT3, and Wnt/*β*-catenin axes in the ovarian cancer microenvironment, inducing high expression of Snail/Slug/Twist and driving transcriptional silencing of E-cadherin. Roque et al. (2020) indicates that EMT involves epithelial cells losing intercellular adhesion and acquiring mesenchymal characteristics, thereby enhancing migration and invasion. ISL effectively suppresses invasion and metastasis in ovarian cancer cells by regulating EMT-related signaling pathways. It downregulates expression of mesenchymal markers such as N-cadherin and vimentin while upregulating epithelial markers like E-cadherin, thereby reversing the EMT process (Dai et al., 2023). This mechanism is partially attributed to ISL’s inhibition of the PI3K/AKT/mTOR and MAPK signaling pathways, which play central roles in EMT regulation. By blocking these pathways, ISL reduces the expression of EMT-associated transcription factors such as Snail, Slug, and Twist, thereby suppressing the migration and invasion capacity of ovarian cancer cells (Hosseinirad et al., 2025).

Additionally, ISL indirectly influences MMP expression by modulating signaling pathways such as MAPK and PI3K/AKT (Ganesan et al., 2024a), thereby further enhancing its anti-invasion and anti-metastasis effects. These findings indicate that ISL suppresses ovarian cancer cell invasion and metastasis through a multi-pathway, multi-target approach, offering new therapeutic strategies for ovarian cancer treatment. Sajeev et al. (2025) indicated that ISL further inhibits the invasion and metastasis of ovarian cancer cells by suppressing the expression of tumor-associated matrix metalloproteinases (MMPs). MMPs are a class of proteases that degrade the extracellular matrix and play a crucial role in tumor invasion and metastasis (Conlon and Murray, 2019). ISL reduces extracellular matrix degradation by downregulating the expression of key MMPs such as MMP-2 and MMP-9, thereby inhibiting tumor cell migration and invasion. This mechanism is closely linked to ISL’s inhibition of transcription factors like NF-*κ*B and AP-1, which play vital roles in regulating MMP gene expression. ISL’s inhibition of MMPs is also associated with modulation of the transcription factor AP-1. Specifically, ISL reduces the nuclear translocation of c-Jun and c-Fos (Devi et al., 2009), and diminishes the binding capacity of AP-1 to its binding sites within the MMP-9 promoter region. Consequently, MMP-9 expression is inhibited at the transcriptional initiation stage, which ultimately reduces ovarian cancer cell invasion of surrounding tissues and penetration of the vascular basement membrane, thereby lowering the probability of distant metastasis. Dong et al. (2024) confirmed that ISL can reduce BDE-47-induced increases in ROS levels and increase the activity of antioxidant substances (such as SOD, CAT, and GSH), thereby alleviating oxidative stress.

### 4.3 Regulation of the tumor immune microenvironment

TAMs predominantly exist in the M2 phenotype within the ovarian cancer microenvironment, promoting tumor metastasis by secreting CXCL1 to activate the NF-*κ*B/SOX4 signaling pathway (Wang et al., 2021). ISL directly binds to the TLR4 receptor on TAM surfaces, inhibiting activation of the TLR4/MyD88/NF-*κ*B pathway (Lei et al., 2025). This process is accompanied by downregulation of M2 markers CD163 and CD206 (Pe et al., 2022) and upregulation of M1 markers iNOS and TNF-*α* (Xu et al., 2025). Concurrently, ISL blocks the Gas6-Axl signaling pathway in TAMs, reducing expression of EMT-related transcription factors such as Snail and ZEB2 (Yuan Y. et al., 2024). This polarization regulation reduces VEGF and MMP-9 secretion, decreasing tumor microvascular density by 35.6% and reducing the number of peritoneal metastatic nodules by 40.1% in ovarian cancer xenograft models, thereby creating a favorable microenvironment for subsequent immune cell infiltration (Li H. X. et al., 2022). Yang et al. (2019) observed in colorectal samples that reversal of M2 polarization led to a sharp decline in CD68^+^CD163^+^ cell density at the tumor center and infiltrating margin, accompanied by the emergence of iNOS^+^ cell clusters. Tumor-associated IL-10 decreased by 40%, while TNF-α and IL-12p70 increased threefold. At equivalent doses, ISL replicated this pattern in primary ovarian cancer xenografts. Flow cytometry revealed an M1/M2 ratio increase from 0.4 to 2.7, with tumor mass reduction of 52%. Yao J. et al. (2024) found that ISL enhances M1 macrophage polarization in the ovarian cancer-associated immune microenvironment. Following ISL treatment of THP-1-induced macrophages, IL-12 secretion increased while regulatory T cell (Treg) differentiation was suppressed via the JAK/STAT pathway, thereby enhancing antitumor immune responses.

Following polarization reversal, TAMs secrete a gradient of CXCL9/10, drawing CD8^+^ T cells from the stroma into the tumor nest and establishing a “hot tumor” phenotype (Kazakova et al., 2023). Concurrently, PD-L1 expression on TAM surfaces is downregulated, releasing the clamp on TCR activation and boosting IFN-*γ* secretion by 5-fold (Jayaraman Rukmini et al., 2019). CD103^+^CD8^+^ TILs exhibit significantly elevated density in high-grade serous ovarian carcinoma, concentrated in tumor epithelial regions. These cells correlate with a favorable prognosis, though most express PD-1, suggesting potential exhaustion states (Webb et al., 2014). Ye et al. (2019) experimentally demonstrated a Treg-suppression-survival-enhancement pattern in colorectal cancer. Interventions simultaneously downregulating Treg, elevating the CD8/Treg ratio, and promoting granzyme B^+^ CTL vascular sleeve infiltration may reduce ascites tumor burden. The underlying mechanism is twofold. First, ISL activates the negative feedback axis of AMPK-mTORC1, which weakens STAT3 binding to the FoxP3 enhancer region (Wang et al., 2020), thereby releasing the immunosuppressive phenotype of Treg (Putra et al., 2025). Second, ISL enhances H3K4me3 occupancy on the IFNG promoter of CD8^+^ T cells by inhibiting KDM5C (Lee et al., 2022). This maintains the TCF1^+^ stem-like subset and increases the proportion of PD-1^+^Tim-3^−^precursor-exhausted T cells, leading to objective response upon subsequent anti-PD-1 sequential therapy (Martinez-Usatorre et al., 2020). Regarding dendritic cells, blocking the “do not eat me” CD47-SIRP*α* signal enhances cross-presentation efficiency in cDC1 cells, which excel at presenting exogenous antigens (McCracken et al., 2015). This leads to elevated intratumoral IL-15R*α* levels and prolonged survival of memory CD8^+^ T cells (Fiore et al., 2020). Concurrently, it shifted neutrophils from the N2 phenotype to N1, enhancing ROS bursts that formed NETs that encapsulated residual cancer cells, thereby inhibiting metastatic dissemination (Shafqat et al., 2023). Chen et al. (2025) proposed that ISL downregulates ANXA2 expression, reducing its activation of JAK kinases. In ANXA2-overexpressing ovarian cancer tissues, ISL treatment significantly diminished JAK/STAT pathway activity and enhanced cell proliferation inhibition.

**FIGURE 2:**
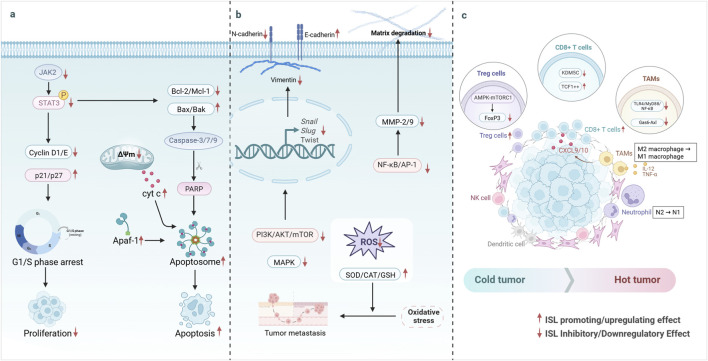
Antitumor mechanisms of ISL in ovarian cancer. **(a)** Inhibition of proliferation and induction of apoptosis; **(b)** Inhibition of invasion ad metastasis; **(c)** Remodeling of the tumor immune microenvironment. Note: Red upward arrows (↑) indicate ISL-mediated promotion or upregulation; red downward arrows (↓) indicate ISL-mediated inhibition or downregulation. Black arrows indicate signal flow or regulatory direction. Dashed lines separate the three subpanels. **(a)** ISL inhibits JAK2/STAT3 signaling, decreases Cyclin D1/E, increases p21/p27, leading to G1/S arrest. ISL also activates the mitochondrial apoptosis pathway (loss of ΔΨm, cytochrome c release, caspase-9/3/7 activation, PARP cleavage) and modulates PI3K/AKT/mTOR and MAPK pathways, while alleviating oxidative stress (increasing SOD/CAT/GSH). **(b)** ISL reverses EMT by upregulating E-cadherin and downregulating N-cadherin, vimentin, Snail, Slug, and Twist. It also suppresses MMP-2/9 activity via inhibition of NF-κB/AP-1, thereby reducing tumor invasion and metastasis. **(c)** ISL remodels the tumor immune microenvironment by promoting M1 macrophage polarization, enhancing CD8^+^ T cell and NK cell infiltration, reducing Treg, and shifting the tumor from “cold” to “hot”. Key mediators include IL-12, TNF-α, CXCL9/10, ROS, and blockade of Gas6-Axl and CD47-SIRPα signals, leading to enhanced antitumor immunity.

The overall immune microenvironment shifted from “cold-suppressive” to “hot-killer,” enabling ISL to synergize with carboplatin and reduce the IC50. Combination with low-dose chemotherapy achieved remission in advanced ovarian cancer mice and established long-term immune memory (Elorbany et al., 2024). ISL modulates the composition and function of tumor-infiltrating immune cells (TIICs) through multiple pathways. On one hand, it increases the infiltration of CD8^+^ T cells and natural killer (NK) cells (Yuan C. L. et al., 2024), which possess direct tumor-killing effects by inducing tumor cell apoptosis through the release of effector molecules such as perforin and granzyme (Díaz Basabe et al., 2021); conversely, it reduces infiltration of Treg and M2-type TAMs (Muñoz Garcia et al., 2021), both cell types typically associated with immunosuppression and tumor progression. Plasma exposure to ISL is extremely low following oral administration; Cao et al. (2020) reported that in BALB/c mice given 35 mg kg^-1^ ISL-SMEDDS, Cmax rose from 0.37 ± 0.12 μg mL^-1^ (free drug) to 1.17 ± 0.35 μg mL^-1^—a 3.16-fold increase; AUC_0-12_ increased from 0.67 ± 0.11 to 2.63 ± 0.55 μg h mL^-1^, with relative bioavailability reaching 392%. Conversely, the half-life shortened from 4.48 h to 3.06 h, indicating that the nanoemulsion bypasses first-pass metabolism via lymphatic transport, rapidly enters systemic circulation, and is cleared quickly, exhibiting a “high Cmax-short t_1_/_2_″profile. This facilitates the rapid onset of action during acute asthma episodes while minimizing accumulation. Song et al. (2022) encapsulated ISL within angiopep-2-modified micelles. Following intravenous administration of 2 mg kg^-1^ in rats, ISL-M’s ${\text{AUC}}_{0-\infty }$ increased to 555.25 ± 60.42 ng h mL^-1^, 1.49 times that of the free drug group; t_1_/_2_ *β* extended from 0.70 h to 1.82 h, and brain AUC_0-2_ h increased 2.58-fold, confirming that the targeted carrier further prolongs circulation time and reshapes tissue distribution. These combined regulatory effects enhance immune surveillance and clearance capacity against ovarian cancer, contributing to the suppression of tumor growth and metastasis (Figure 2).

## 5 Prospects and challenges of ISL in ovarian cancer treatment

### 5.1 Potential of ISL as a monotherapy

Li et al. (2018) demonstrated that 30 μM ISL elevated apoptosis rates to 79.7% and 59.2% in SKOV3 and OVCAR3 models, respectively. The mechanism involved sustained dephosphorylation of p-Akt and p-mTOR, increased GSK3*β* expression, amplified Bax/Bcl-2 ratio, decreased mitochondrial membrane potential, and substantial accumulation of the active Caspase-3-p17 fragment. At the same concentration, colony formation decreased by 40%, migration distance shortened by threefold, and invasion decreased by 60%. Mahalingam et al. (2016) further demonstrated that 36 μM ISL reduced Cyp19a1 and Hsd17b1 transcription in mouse antral follicles by 2.5-fold and 3-fold, respectively, within 96 h. For platinum-resistant ovarian cancer with wild-type BRCA or p53 mutations, ISL bypasses DNA damage repair pathways to directly trigger mitochondrial apoptosis, offering a novel “de-chemotherapy” maintenance option (Huang et al., 2024). Liu et al. (2023) demonstrated that ISL induces cell cycle arrest, apoptosis, and autophagy in uterine leiomyoma cells while inhibiting estrogen-induced extracellular matrix (ECM) accumulation and related protein expression, thereby regulating the MAPK signaling pathways. This significantly suppressed fibroid cell proliferation, reduced ECM deposition, and decreased *in vivo* fibroid weight and hormone levels, demonstrating therapeutic potential. Tastan et al. (2022) found that ISL significantly reduced ovarian cancer cell proliferation, with experimental data showing a roughly 40% decrease in proliferation after treatment.

### 5.2 Combination therapy with ISL and other treatments

Combining ISL with mTOR inhibitors significantly enhances growth inhibition of ovarian cancer cells by dual blockade of the PI3K/Akt/mTOR pathway (Li et al., 2018; Zhang F. et al., 2022). Qiao et al. (2020) prepared ISL nanosuspensions (HPC SSL-ISL-NS and PVP K30-ISL-NS), which can improve drug solubility, achieving 75%–90% dissolution rates within 20 min in pH 1.2 and pH 6.8 media, significantly higher than that of the free drug (32%). This nano-suspension exhibits higher cytotoxicity against A549 lung cancer cells, with a mechanism potentially applicable to ovarian cancer. When combined with chemotherapeutic agents, the nano-suspension’s high solubility and cellular uptake capacity (fluorescence intensity of HPC SSL-C6-NS uptake significantly exceeded that of free drug) can enhance local tumor drug concentration, boost chemotherapy efficacy, and reduce both drug dosage and toxic side effects. Jacob et al. (2025) further explored the application of solid lipid nanoparticles (SLNs) and nanostructured lipid carriers (NLCs) in delivering anticancer phytochemicals, noting these nanocarriers protect ISL from degradation while enabling controlled release and targeted delivery. Sm et al. (2022) reported that plant-derived alkaloids can significantly enhance their antitumor effects when delivered via nanomedication. After encapsulation in nanocarriers, ISL exhibits improved bioavailability, enabling greater penetration into tumor tissues and enhanced inhibition of the JAK/STAT pathway. Nourbakhsh et al. (2023) demonstrated that combining ISL with chemotherapy drugs like cisplatin significantly enhances the inhibition of ovarian cancer cells. This combination exerts effects through multiple pathways: ISL suppresses the JAK/STAT signaling pathway, reducing the production of inflammatory factors and thereby degrading the tumor cell microenvironment; simultaneously, chemotherapy drugs such as cisplatin directly damage tumor cell DNA, inducing apoptosis. Their synergistic interaction markedly enhances cytotoxicity against ovarian cancer cells. Xiang et al. (2018) demonstrated that ISL inhibits melanoma growth by downregulating miR-301b, thereby inducing LRIG1 expression; LRIG1 is also frequently underexpressed in ovarian cancer. This suggests that when combined with immunotherapy, ISL may enhance tumor immunogenicity by regulating the miR-301b/LRIG1 pathway, thereby improving the efficacy of immune checkpoint inhibitors and providing a new target for combined ovarian cancer therapy.

### 5.3 Challenges and prospects for clinical application of ISL

ISL, characterized by multi-pathway regulation, low toxicity, and broad-spectrum antitumor activity, effectively suppresses malignant proliferation, invasion, and metastasis of ovarian cancer cells by targeting and inhibiting aberrant activation of the JAK/STAT signaling pathway. It also reverses the immunosuppressive tumor microenvironment and sensitizes chemotherapy. Thus, ISL provides a new natural candidate for targeted intervention, reversal of drug resistance, and combination therapy in ovarian cancer, holding significant value for basic research and clinical translation.

At present, the clinical application of ISL still faces several bottlenecks. First, drug formulation limitations: ISL has poor water solubility, significant first-pass metabolism, a short half-life *in vivo*, and insufficient tumor-targeting accumulation, making it difficult to maintain effective antitumor concentrations through conventional administration. Second, research system limitations: most existing studies rely on *in vitro* cell experiments and small-scale nude mouse models, lacking high-level animal studies and clinical sample validation. Third, fragmented mechanistic studies: current research mainly focuses on single metabolic or inflammatory pathways, without systematic evaluation of pathway crosstalk, dose-dependent toxicity, or long-term safety.

There are still critical knowledge gaps in this field. The differential regulatory mechanisms of ISL on the JAK1/2/3-STAT1/3/5 signaling axes in the context of ovarian cancer remain unclear. Few studies have investigated the relationship between ISL-mediated JAK/STAT regulation and EMT, the ascites microenvironment, or peritoneal dissemination in ovarian cancer. Moreover, the synergistic targets and optimal dosing regimens for combining ISL with JAK inhibitors, platinum-based chemotherapy, and immune checkpoint inhibitors have not yet been elucidated. Future efforts should focus on improving bioavailability through nano-targeted delivery technologies and exploring the cross-regulatory mechanisms of the JAK/STAT core pathway, thereby advancing ISL from basic research toward clinical adjuvant therapy for ovarian cancer.
